# “The role of the man is to look for food”: Lessons from men’s involvement in maternal and child health programmes in rural Central Malawi

**DOI:** 10.1371/journal.pone.0221623

**Published:** 2019-08-23

**Authors:** Elizabeth Mkandawire, Sheryl L. Hendriks

**Affiliations:** 1 Institute for Food, Nutrition and Wellbeing, University of Pretoria, Pretoria, South Africa; 2 Department of Agricultural Economics, Extension and Rural Development, University of Pretoria, Pretoria, South Africa; Yenepoya Medical College, Yenepoya University, INDIA

## Abstract

Many studies purport that in low-income countries, women are often responsible for producing, preparing and purchasing food. Consequently, policies related to food and nutrition overemphasise the role of women, underestimating the potential for cooperation and complementarity between men and women. This focus on women does not account for socially constructed expectations of women that undermine their decision-making in resource allocation. Using desk reviews, in-depth interviews and focus group discussions, our case study in Malawi sought to understand the complementary role of men in maternal and child nutrition. International agreements and Malawi’s policies were reviewed to understand how men’s involvement emerged on the nutrition policy agenda. Policymakers, stakeholders and men and women from rural Central Malawi were interviewed, sharing their experiences of men’s role in maternal and child health. The study found that men’s involvement in maternal and child health has been on the development agenda since as early as 1995. Malawi has made efforts to involve men in these areas through several policy actions and programmes. Contrary to literature suggesting that women are the main producers, procurers and preparers of food, this study found that men in rural Central Malawi are increasingly becoming responsible for providing and purchasing food. Men also play a supportive role in food preparation, helping women access diverse diets during and after pregnancy. They also take up a supportive role in household activities, providing women with assistance in housework and looking after children. The positive change in men’s roles presents an opportunity for exploring how men can contribute to food security and nutrition. Opportunities exist for designing inclusive food and agriculture policies that promote cooperation between men and women in food and nutrition. These policies can challenge misinterpretations of women’s role in food security and the underlying systems that reinforce gender inequalities.

## Introduction

Based on a 1997 FAO report that focused on women and food security [1], many studies have emphasised the role of women in food security, purporting that women are responsible for much of the world’s food production [2]. More recently, the 2011 FAO State of Food Security and Agriculture report suggested that if women had the same access to resources as men, they could increase yields on their farms by 20–20% [3]. However, targeting women alone does not challenge gender power relations such as household decision-making, that constrain women’s access to resources for improving productivity. For example, a case study by Djurfeldt et al. [4] suggests that even when women have access to and control over land, men are still the primary decision makers in the home. While increased access to land could improve women’s productivity, policies rarely compliment women’s land rights with a genuine understanding of the gender relations that undermine women’s productivity.

Three underlying truths challenge the misconception that women are primarily responsible for the world’s food production. The first is women’s time burden. Several studies [3; 5; 6] suggest that women’s productive time is constrained by the roles involving unpaid care and housework that society expects them to play. Such constraints hinder women’s productivity. Gender role expectations such as women’s responsibility for childcare prevent them from having enough time to participate in agricultural activities. Women face difficulties in balancing their care and domestic responsibilities with agricultural work. For example, when a family member becomes ill, women are expected to look after them. This means that they lose time which they could otherwise spend on the farm.

The second truth is that women’s limited access to resources and services undermines their productivity. Women are consistently found to be less productive than their male counterparts because of limited access to labour, land, knowledge, fertiliser and improved seeds [3; 7; 8; 9; 10]. Women’s productivity remains constrained even when access to resources is increased because the underlying factors that constrain women’s access to these resources are not addressed. While women’s limited access to resources does indeed cripple their productivity, other factors such as women’s control over household resources need to be considered in policy design.

The third truth is the fact that women’s labour in agriculture cannot be separated from men’s labour. No evidence exists to support the overstated claim that women produce 60% to 80% of the world’s food [2]. One of the main challenges in providing evidence for this statistic is that it is difficult to measure the labour productivity of men and women separately when agricultural labour is often carried out jointly by families [11; 12].

If women are constrained by time and resources and men’s labour is not accounted for in food production, is it plausible that women can produce half of the world’s food? The implications of such misconceptions for policy design are concerning when these underlying truths are unpacked. The emphasis on women increases the expectation of and burden on women in low-income countries [11]. Women already face increased time constraints owing to socially determined responsibilities for housework, care work and indeed the production of household food [13]. Furthermore, these misconceptions do not account for gender roles and relations which systematically constrain women’s ability to provide nutritious food for themselves and other family members. Such nuances often do not reach policymakers and can lead to an overemphasis on women resulting in a bias in the design of food and nutrition policies.

The misconceptions around women’s role in food security are also inconsistent with changing gender dynamics. Gender roles and relations vary from one country to another and even within the same culture. However, one similarity exists across the globe: gender roles are not static and can change as the needs of society change. The increase in women’s education, employment and participation in the economy are some of the factors that contribute to shifts in gender roles [14]. While shifts in gender roles are more pronounced in European countries [15], changes in African countries are also being observed. These changes have implications for how gender is integrated into policy.

Mkandawire et al. [16] suggest that in Malawi, men are increasingly taking on responsibility for housework and care work because of initiatives that are driven by the health sector. Non-Governmental Organisations (NGOs) including Concern, Care Universal Malawi and Catholic Relief Services have implemented interventions to encourage men to participate in maternal and child health in Malawi. Concern Universal implements several men’s involvement in gender equality interventions based on evidence that affirms the importance of men’s participation in gender equality [17]. Care International has developed a range of men’s involvement in gender equality interventions in the various countries in which the organisation works. Catholic Relief Services has also been working in several low-income countries to integrate men’s involvement initiatives in their programming, basing these interventions on the premise that the active participation of men and boys makes interventions more meaningful and sustainable [18]. While there is a need for broader quantitative studies on the outcomes of men’s involvement in maternal and child nutrition, several studies indicate that men’s involvement in maternal and child health can be positively associated with maternal nutrition [16; 17; 18; 19; 20; 21].

These interventions are changing the relationships between men and women, indicating that attention to women alone overlooks the possibility of cooperation and complementarity between men and women [11]. The change in gender dynamics has implications for food and nutrition policies.

Drawing on the reported experiences of study participants in Malawi, this study sought to investigate how men’s involvement in maternal and child health interventions are changing the role of men in household food production, preparation and purchasing, emphasising the implications of changing gender dynamics for food security and nutrition policy design. The study also aimed to highlight the positive role men can play in advancing gender equality and improving food security and nutrition outcomes.

### Setting the scene–mainstreaming men in Malawi’s food and nutrition policies

**Fig 1:**
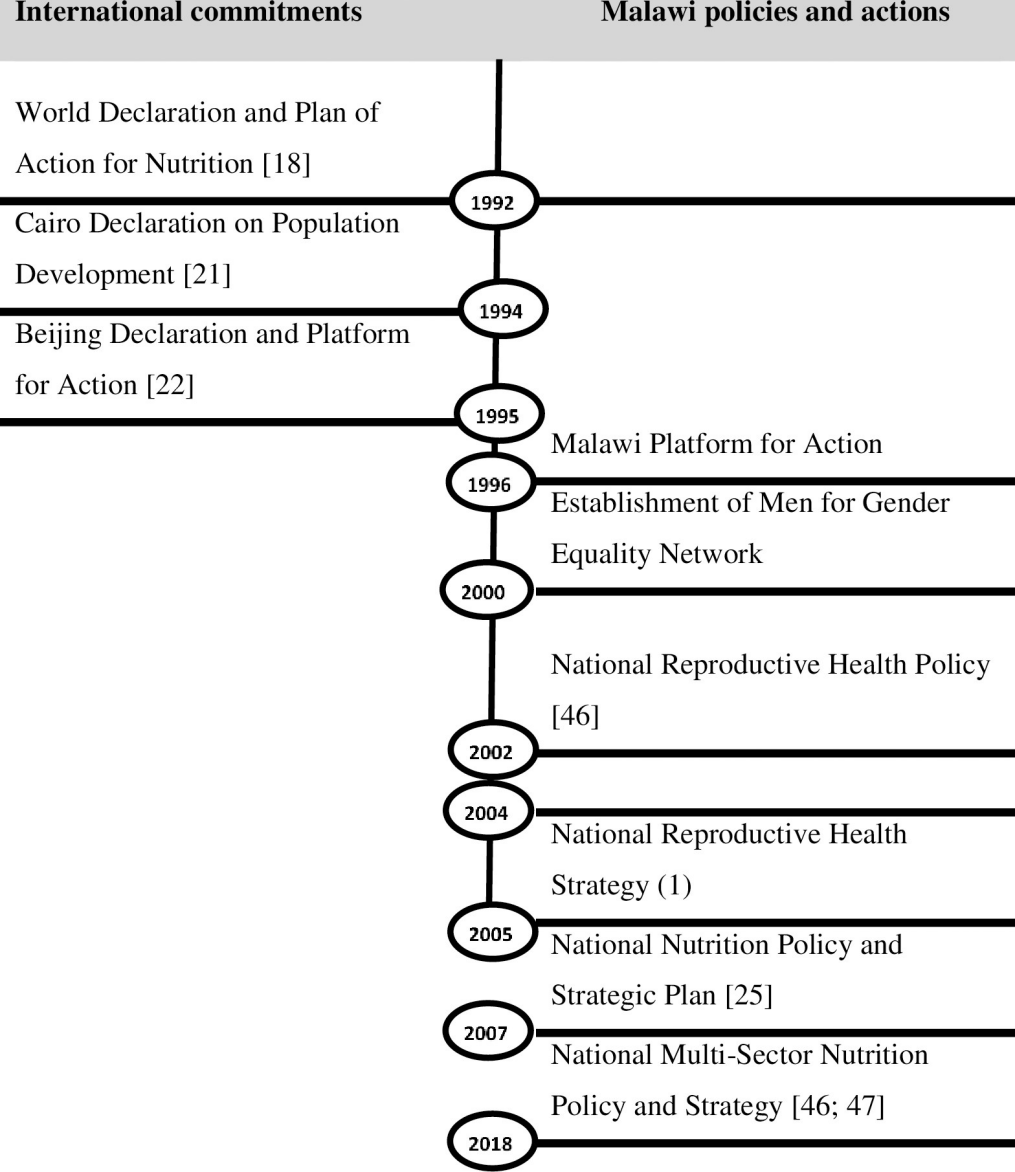
Policy timeline with gender equality and nutrition outcomes.

While the following section is not a traditional literature review, it provides an overview of the policy landscape related to men’s involvement in gender equality in Malawi. In order to understand how the role of men in gender equality is integrated into Malawi’s health and nutrition policies, a review of international, African, regional and national agreements, policies, strategies and legislation was conducted. Fig 1 presents a timeline of these events and agreements.

In Malawi, traditional gender roles often placed responsibility for child care, housework and food in the hands of women. From as early as 1949, women have been responsible for childcare, which often impacts their ability to be mobile during periods of food shortage. Men on the other hand could migrate or work for food or cash. In the past, women’s participation in care work, house work and farming, has meant that they have a greater workload than men. The multiple roles and responsibilities often prevented women from pursuing other activities to help improve their economic status. The Malawi Labour Force Participation Survey clearly demonstrated this in 2014 through a report that showed how women’s time spent on unpaid care work was six times higher than that of men [22]. While it is important to note that variations of women’s time burden may exist across matrilineal and patrilineal societies in Malawi, socially prescribed gender roles remain consistent across the country [23].

The more recent literature on gender roles in Malawi has focussed on men’s role or lack thereof in pregnancy and childbirth. Men have often regarded pregnancy, childbirth and by extension childcare as women’s domain [24]. Consequently, women have authority and decision-making power within these domains. Mbweza et al. [25], suggest that gender roles indicate specific domains in which men and women have authority. Efforts to involve men in areas that have typically been controlled by women should support joint decision-making to ensure that women’s authority is not appropriated by men [26, 27, 28]. The trends in global development spaces around men’s involvement in maternal and child health have influenced efforts in Malawi to encourage men to participate in these domains [29].

The role of men in maternal and child nutrition was highlighted as critical in as early as 1992 during the First International Conference on Nutrition [30]. At this conference, the 1992 World Declaration and Plan of Action for Nutrition was signed by UN member states, highlighting that men’s control over resources determined nutrition outcomes. In 1993 development focus shifted from the ‘Women in Development’ approach to the ‘Gender and Development’ approach [31]. This shift was made because development practitioners realised that a focus on women alone was not enough to challenge the systemic factors that undermine women’s equality [32]. Malawi embraced the concept of gender as early as 1994, by signing the Cairo Declaration on Population Development which emphasised “…eliminating negative gender stereotypes in order to improve the status of women, with a view to achieve cooperation and partnership between men and women” [33]:3. Central to this agreement was the understanding that as the primary household decision-makers, men controlled the resources needed to unlock positive outcomes in women’s sexual and reproductive health.

In 1995, Malawi signed the Beijing Declaration and Platform for Action [34]. This Declaration committed all member states to integrate gender in all development actions including policies and programmes, and all levels. In particular, section 179(c) commits UN member states to “Ensure, through legislation, incentives and/or encouragement, opportunities for women and men to take job-protected parental leave and to have parental benefits; promote the equal sharing of responsibilities for the family by men and women, including through appropriate legislation, incentives and/or encouragement…”

These two declarations emphasised addressing power relations between men and women, highlighting cooperation between men and women as the foundation for achieving development objectives. Malawi integrated these agreements in development planning in 1996 by adopting the Malawi Platform for Action [35]. In integrating these agreements into various areas of development, Malawi was one of two countries in the Southern Africa Development Cooperation (SADC) region to include a chapter assessing men’s participation in health care in the 2004 Malawi Demographic and Health Survey. Lesotho was the only other country in the SADC region to include this chapter. Malawi’s 2006 Reproductive Health Strategy [36] also included a chapter on men’s involvement in reproductive health. Men were identified as a special group, highlighting that existing reproductive health services were not male-friendly. According to the strategy, an activity that had been initiated during this time to address this concern was a ‘male championship’ project in Mwanza which was yet to be rolled out. The strategy set out specific targets for increasing the number of men accompanying their partners to antenatal clinics as well as increasing the number of men who knew the signs and symptoms of pregnancy complications [36].

Malawi’s Sexual and Reproductive Health and Rights Policy was adopted in 2009 [37]. The policy included a specific goal to promote men’s involvement in sexual and reproductive health and rights issues and services. Emphasis was placed on men’s shared responsibility in parenthood and sexual and reproductive health. In August of the same year, the Centre for Reproductive Health in collaboration with MoH hosted the Second Reproductive Health Conference [38] where issues of men’s involvement were highlighted as central to achieving positive maternal and child health outcomes in Malawi. Four key issues were highlighted in respect of men’s participation in maternal and child health:

- Participation of traditional leaders in encouraging men to be involved in maternal and child health
- Need for clear guidelines and policy on the levels of male involvement
- Need for male-friendly infrastructure in labour wards where women give birth and
- Need to address socio-cultural myths, barriers and misconceptions around men’s involvement in maternal and child health [38].

The Malawi Growth and Development Strategy III (MGDS III) [39], the country’s national development plan, mentions men’s involvement in nutrition. In the same year, the National Multi-Sector Nutrition Policy and Strategy were passed, both emphasising men’s participation in housework and childcare to enable women to have more time to pursue economic and social activities [40; 41].

### The research context

Malawi is a land-locked country located in Southern Africa with a total estimated population of 17.6 million people [42]. The country is divided into three regions: Northern, Central and Southern regions. These regions are further divided into 28 districts, which are subdivided into traditional authorities [43]. Traditional authorities have political oversight over all the chiefs in their jurisdiction. Group village headmen/women are responsible for eight to fifteen chiefs’ who oversee villages consisting of 15 to 40 families [44].

In Malawi, traditional leaders play an increasingly important role in the implementation of policies, particularly the implementation of policies within the Ministry of Health. In collaboration with the district health office, traditional leaders have established bylaws encouraging women to stay at maternity waiting homes (MWH) in their eighth month of pregnancy [45]. Maternity waiting homes are structures that are close to a health facility and were established as one of the conditional recommendations of the WHO. These MWH were also established as part of the Presidential Safe Motherhood Initiative launched in 2012 to reduce infant and maternal mortality [46; 47].

Malawi’s agriculture sector accounts for one-third of the country’s gross domestic product and employs 80% of the productive labour force [48]. While the agriculture sector has seen increased investment because of the Comprehensive African Agriculture Development Programme (CAADP) [49] and commitments such as the Malabo Declaration on Accelerated Agricultural Growth and Transformation for Shared Prosperity and Improved Livelihoods [50], persistent shocks in the form of climatic events have frustrated efforts to improve agricultural productivity. Even moderate droughts have had a severe impact on food security for families in rural Malawi [51].

While erratic weather patterns have exacerbated food insecurity in Malawi, stunting in children under five reduced from 47% in 2010 [52] to 37% in 2015 [43]. However, Malawi still has one of the highest rates of stunting in children under five [53]. Only 8% of children access the minimum acceptable diet of at least four food groups per day [44].

The World Bank projects that 70% of Malawi’s population was living below the international poverty line of less than $1.90 per day in 2016. With repeated weather shocks, this rate is expected to have increased [54]. Many Malawians are involved in *ganyu* as a means of generating income. Dimova et al. define *ganyu* [55] as off-farm, informal labour. After own-farm production, *ganyu* is the most important source of livelihood for poor households in Malawi [56]. *Ganyu* is often used as a coping strategy after shocks and as an insurance measure before shocks [57].

### Study location

This study was conducted in the Central Region of Malawi in Ntcheu District. The total estimated population of Ntcheu is 592,895 [42]. In this district, 84% of women were employed in agriculture compared to 15% of men in 2015. Many of these women reported being engaged in informal, seasonal employment. The majority of men (61%) were employed in unskilled manual services. Of the 36% of women and 37% of men who owned land in Ntcheu, 98% of both men and women did not own a formal title deed [43].

In Ntcheu district 62% of men and women reported joint decision-making over the wife’s earnings. In terms of men’s earnings, 39% of men compared to 72% of women reported joint decision making over men’s cash earnings [43]. Women in Ntcheu reported the highest incidence of joint decision-making over men’s cash earnings compared to other districts in Malawi. Independent or joint decision-making of married women in the age cohort 14–59 years regarding women’s health, household purchases and visits to wife’s relatives increased from 21% in 2010 to 73% in 2015 reported [43].

## Methodology

This study set out to understand how men’s increased participation in maternal and child health is re-shaping gender dynamics in Ntcheu district. The study also aimed to investigate the implications of these changes for food and nutrition policy. A desk study was conducted to understand how men’s involvement is integrated into food and nutrition policies in Malawi. The previous section documents the historical progression of the integration of men in gender equality approaches. The section highlights Malawi’s current policy position on gender equality in nutrition policy.

The following section applies qualitative research methods using a critical policy framework. Men and women from one community and Health Surveillance Assistants (HSA) were interviewed. The HSAs were included in the study to corroborate research findings from the interviews with the men and women as well as deepen our understanding of how gender dynamics have changed in this community.

### Data collection

#### Focus group discussion

Five focus groups discussions (FGD) were conducted with men and women in rural central Malawi. The FGDs elicited varied opinions from the participants and allowed for validation where conflicting opinions emerged. The discussion lasted between one to two hours. The discussions were conducted at the maternity waiting homes or community gathering spaces after large groups had left. For example, at the postnatal mobile clinic, we waited for the health surveillance assistants to finish their activities and dismiss the attendees before we commenced with the FGDs.

The study applied purposive sampling to identify key informants such as the HSAs. The District Health Office referred us to eight HSAs. All eight HSAs responsible for the targeted community were interviewed. The HSAs assisted us in identifying men and women participants at the mobile postnatal clinics where they conducted their work. At the maternity waiting homes, the nurses helped us to identify participants. Convenience sampling was then applied in identifying other respondents depending on their availability. The traditional authority assigned us a key informant who eased access to community members volunteering to participate in the discussions. A total of 36 participants were interviewed. The majority of participants were women (*N = 22)*. We were concerned that men might have overstated their involvement in housework and care work in the first FGD with men. More women were thus sampled in the FGDs to verify and corroborate men’s involvement in these activities. One FGD was conducted with men, three with women and one was conducted with both men and women. The mixed-sex focus group discussion was conducted with the HSAs.

Mixed-sex focus group discussions that included men and women participants were necessary to understand the gender perspectives. Having both men and women in focus group discussions enabled us to observe the interactions between men and women. These discussions were only held with the HSAs who are trained on issues of gender. Although this discussion was skewed in favour of the men because of the number of male HSAs in this community, women still responded 21 times compared to men who responded 31 times. The number of responses was counted to determine equal participation between men and women in the discussion.

The discussions were conducted in Chichewa, by one of the authors and an experienced facilitator. Both are fluent in Chichewa. One of the facilitators is experienced in qualitative research and was able to create trust and rapport amongst participants. The lack of a male facilitator for conducting FGDs with the men is recognised as a limitation. The men, however, were open in the discussion and seemed to share their experiences without constraint.

#### Ethical clearance

The study was approved by the research ethics committees of the two institutions involved in the study. In Malawi, clearance to conduct the study was obtained from the Ministry of Agriculture Irrigation and Water Development. The District Commissioner provided written consent for the research to be conducted in the area. The traditional authority of this area, as well as the chiefs in each village, were also consulted prior to data collection and permission was granted to proceed with the study.

The Health Surveillance Assistants all signed written consent forms. Community members’ consent was recorded digitally because of the researchers’ concerns regarding limited formal education of the community members. Each participant was given a number. The participant was asked to state their number and confirm that they consented to participate in the study. The FGD facilitators explained that participation was voluntary and that respondents could withdraw from the study at any time without any repercussions. Participant’s confidentiality was ensured by anonymous data entry.

### Data analysis

**Fig 2:**
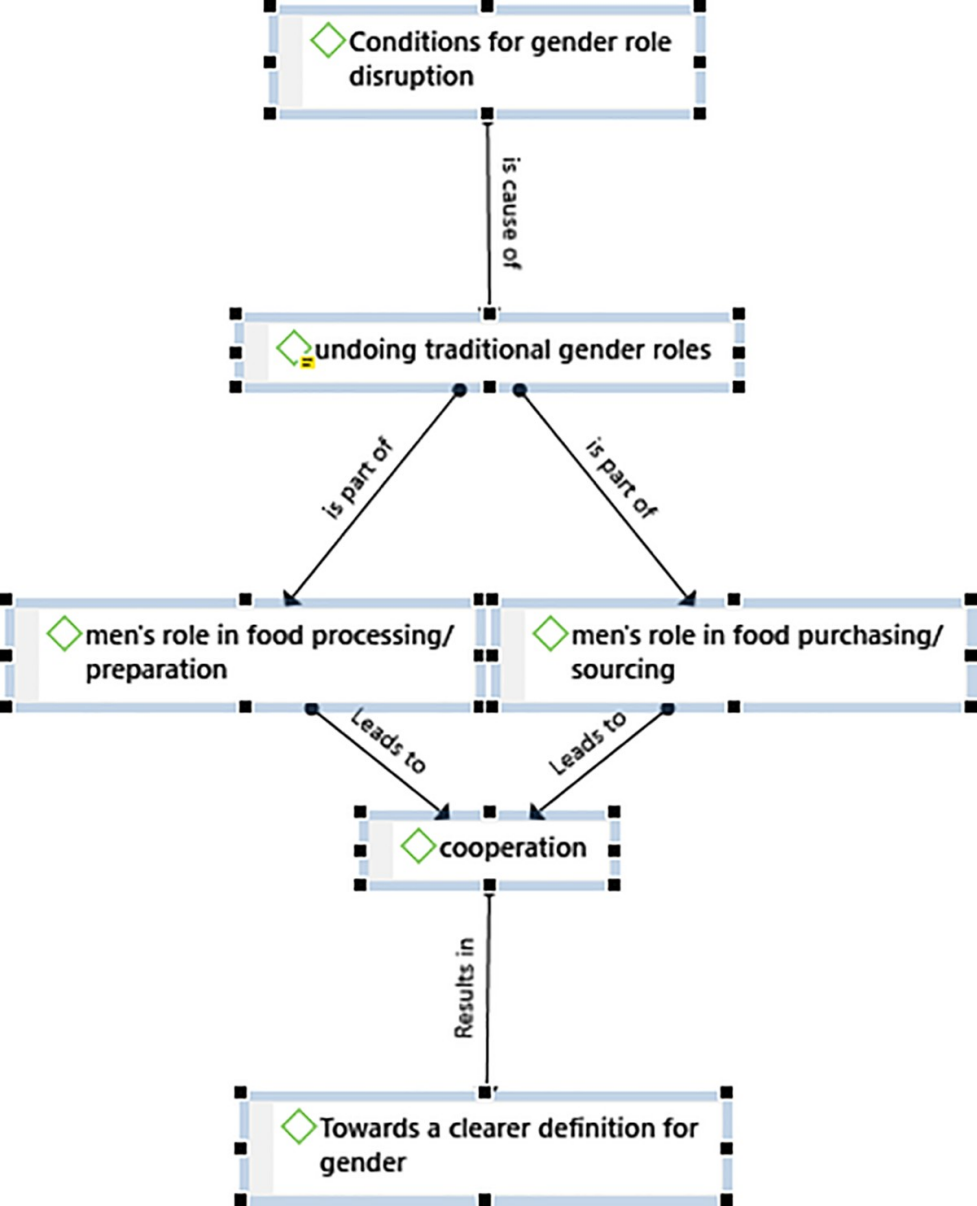
Themes identified for changing gender dynamics and how they relate to one another.

Focus group discussions were audio recorded. All five FGD were transcribed, and Chichewa transcripts were translated into English. The transcripts were uploaded onto Atlas-*Ti* and reviewed. The first author was involved in the data analysis. Ten themes were identified and quotations presenting each of these themes were captured. These themes were selected based on patterns that were identified during the review of the transcripts. If certain themes had less than three quotations, they werenot included. This assisted in reducing biases based on the interests of the researcher. The themes were then revised, and some were merged, while others were split into sub-themes. Six themes formed part of the final codes. To reduce subjectivity, these themes were reviewed and revised by the second author and another researcher who had participated in data collection. Once the coding was completed, a report was produced allowing us to organise the results and write-up the research findings. Atlas-*Ti* was also used to create a network which provides a summary of the research results. Fig 2 below illustrates and summarises the research findings.

## Results

Demographic data were collected for all the community member participants. The mean age of the participants was 37, ranging between 19 to 80 years old. The average level of schooling completed was standard seven for women and form two (or grade nine) for men. The level of schooling ranged between participants who had no formal education to participants who had completed their secondary education. All of the participants were involved in subsistence farming and *ganyu* labour to sustain their livelihoods.

The results indicated that men’s participation was indeed shifting gender dynamics and that a new definition of gender is emerging in this community. Fig 2 maps out the progression of themes indicating how *conditions for gender role disruption* result in more flexible gender roles. The *undoing* of *traditional gender roles* includes men taking up *roles* in *food processing/production* and *food purchasing or sourcing*. The transition from strictly adhering to traditional gender roles to more flexible gender roles has resulted in greater *cooperation* between men and women and consequently a *new definition of gender* is emerging in this community. The themes are mapped out in this order to illustrate how they relate to one another, but also to indicate how the positive roles that men are taking up are increasing cooperation and advancing gender equality.

### Conditions for gender role disruption

The study found that while men did indeed take on responsibilities that have typically been associated with women, they only did so under certain conditions or circumstances. Government interventions are leaving men with no alternative but to take on work that is typically associated with women. For example, the bylaws passed by the traditional authorities meant that many women have to stay in the maternity waiting homes in their eighth month of pregnancy. Some women are escorted by a guardian (usually a mother or sister) who remains with them until the child is born. As a result of this bylaw, the mother is forced to leave her children at home. In a focus group discussion with women at the maternity waiting homes, one woman mentioned that men help when women are at the maternity waiting homes:

“When you are pregnant, he does help. Like now that I am here, my husband is the one who is looking after the other children.”

Five of the eight women interviewed at the maternity waiting homes reported that their husbands helped them when they became pregnant. However, this was not the case with all the respondents. One woman mentioned that she was not as comfortable with the idea of her husband remaining at home with the children. She was concerned about leaving her children at home. She said:

“You stay here with worry. Like I came on Monday. I have left a two year old at home. I’ve also left an eleven year old…And the father doesn’t do things properly.”

While advocacy, through avenues such as radio messages encouraged men to support their partners during pregnancy, not all men were supportive. One woman in a focus group discussion at the maternity waiting homes said:

“Like my husband, it goes in one ear and out the other. I started from the first month to the ninth month complaining about my back, but he has not even tried to light the fire…no!”

Some women expressed that men also show a willingness to help when women are busy. When asked if men really do help with household chores, another woman in a focus group discussion at the postnatal mobile clinic mentioned that when men realise that women are busy, they help with household activities:

“Yes. They can cook and wash when they see that we are very busy.”

Several respondents expressed that men also help with housework and childcare when women are sick. A woman in the focus group discussion with women at the postnatal clinics mentioned that sickness is another reason which prompts men to assist with household activities.

“He helps me with the chores when he sees that I am sick.”

### Undoing traditional gender roles

Although traditional gender roles still prevail in rural Central Malawi, there is evidence of a shift towards more relaxed gender roles, where men and women are becoming increasingly involved in activities that were previously associated with a single sex. For example, when asked if men help with household chores, one woman respondent from the postnatal clinics expressed that:

“Some of them go to fetch water, and some do not.”

Many of these shifts are an outcome of gender advocacy from various NGOs, and it is evident that both men and women are taking up roles that were previously associated with the opposite sex. One male Health Surveillance Assistants reported that:

“Concern [an NGO working in this district] was also advocating for gender before it phased out. Women were encouraged to do jobs that men would also do.”

While in the past, men have resisted participating in work that was typically associated with women, men are increasingly becoming more open to supporting their partners. One man from a focus group discussion said:

“So even now we go together with our wives to the maize mill, and that does not mean you are a fool.”

Both men and women reported that men were involved in many activities that have traditionally been associated with women. Two specific areas of work were identified: men’s role in food processing/preparation and men’s role in food sourcing/purchasing.

### Men's role in food purchasing/sourcing

Men in this community played an important role in purchasing and looking for food. In fact, both men and women reported that looking for food was a man’s responsibility. One woman from a focus group discussion with women said:

“It is the responsibility of the man to be buying the required things at home for the family to remain healthy.”

When asked if men help with household responsibilities, one woman in the focus group discussion responded to say:

“Yes, it is his responsibility too! The role of the man is to look for food.”

The men also expressed that purchasing and looking for food was indeed their responsibility. One man in a focus group discussion said:

“As for me, when the maize that I have will be finishing I will have to ask for piece work from someone who is having it so that he or she should pay me maize. So I will work for seven days, and then I will be given about one pail of maize. Then I can give it to my wife who will go with it to the maize mill and then we can eat at home.”

Two other men also shared that they look for *ganyu* in order to provide food for their families. The men mentioned that *ganyu* is usually sought after when the food harvested is not enough to make ends meet. Men reported working for food as well as working for cash to buy food.

### Cooperation

Both men and women mentioned that gender equality advocacy had increased cooperation between men and women. One woman in a focus group discussion with women expressed that:

“…we are now seeing things changing. Because even now we say at least let me cook the relish, so that when I leave, at least he can cook the *nsima* (a stiff porridge made from maize meal). But these things are changing now. In the past, it was not like this.”

The Health Surveillance Assistants also expressed that men and women were working together to ensure that their families had food and were healthy. Men and women share child caring responsibilities. One male Health Surveillance Assistant mentioned that:

“There are times when a woman is busy and cannot go to the hospital with the child. It is then the man who takes the child to the hospital.”

When asked if women are also involved in *ganyu* labour, one man in the focus group discussion with men said:

“Yes, they help. If they find piece work, we help them, and if we find work, they also help us. So we work together.”

The notion of working together came up in all five focus group discussions. Men and women attend antenatal clinics together as a result of the bylaws established. While some women mentioned that the final decision concerning farming rests with their husbands, many men and women expressed that they worked together with their partners on the farm and to provide food for their families. One woman in a focus group discussion at the maternity waiting home said:

“We work together with our husbands in order to make ends meet. If we see that the food, we have harvested is not enough, we make decisions together with our husbands to say that we should buy some food to top up.”

One woman in the women’s focus group discussion mentioned that even in terms of decision-making, men and women work together:

“There are some men who do understand and respect their wife’s ideas but others not. You can discuss with each other on how to farm for the year. We decide on how many hectares to farm together. Even when it comes to fertiliser, you decide together how many bags to buy.”

### Towards a clearer definition of gender

With the blurring of gender roles and increased cooperation between men and women, a new understanding of the relationship between men and women is evident in this community. One woman in a women’s focus group discussion said:

“With the issue of gender, there is no difference between the man and the roles of the woman. You are both expected to work the same way. The difference which is there is that the man is the head of the family the woman not. But when it comes to work, it is important that both should help. When the woman is busy with the child, the man should help to cook the food.”

Gender is understood in this community as working together. One male HSA in a focus group discussion said:

“Gender means equality between men and women apart from the things provided by nature. It is natural for women to give birth. There is no way a man can give birth in the name of gender.”

Another male HSA said:

“There is a need for gender. Men and women must work together.”

The men in the focus group discussion concurred that when their partners are pregnant, they help them with cooking, fetching water and farming. One man expressed that:

“Initially things were not like this, these days there is gender [equality].”

## Discussion

The review of existing policies on men’s involvement in Malawi indicates that the health sector in has implemented efforts to involve and harness the complementary role of men in health and nutrition. The study respondents confirmed that the men’s involvement in maternal and child health interventions have had an influence on gender dynamics in the community. The reported changes in gender roles reflect the perceptions of the focus group discussion participants and do not reflect men’s past roles in nutrition. Men may have indeed participated in nutrition in the past, however, the degree of participation, cooperation and gender roles may have changed over time. The 2015 Malawi Demographic and Health Survey reports that joint decision-making between men and women in the sampled community increased by 52 percentage points between 2010 and 2015 [42]. The respondents indicated that there are certain conditions under which men take on responsibilities that have typically been associated with women. When women are busy, sick or pregnant, men take on more responsibility for cooking, cleaning and childcare. Specific government interventions and strategies have contributed to increasing pressure on men to take on work that has typically been associated with women. These interventions may have influenced the ways in which men and women currently conceive and articulate participation.

In the eighth month of pregnancy, when women are expected to stay in the maternity waiting homes, some women reported that men take care of the children who remain at home. The circumstances necessitate a substitution in gender roles and responsibilities–only if no other women step in to look after the children while the mother is away. These interventions have contributed to fostering cooperation between men and women. While it is not in all instances that men are actively involved in child care, policymakers can leverage the positive roles men are playing to improve the gender equality at the household level.

Men participate in food preparation as well as looking for food. While many studies indicate that food production, purchasing and preparation are women’s responsibility, both men and women participants in this study concurred that these are actually men’s responsibility. In fact, men explained that they look for *ganyu* to ensure that they can provide food for their families during periods of low harvests. Men are also increasingly playing a role in food preparation. Several men and women expressed that cooking is a shared responsibility between men and women.

Nutrition policies often target women as the primary beneficiaries of policy interventions particularly in relation to nutrition education because studies have frequently reported that women are the primary producers, preparers and purchasers of food. The results presented in this paper suggest that policymakers need to re-think how policies are designed and consider the changing gender dynamics that ensue as an outcome of policy interventions from other sectors.

Interventions encouraging men to participate in maternal and child nutrition and health are increasing cooperation between men and women, even in what are assumed deeply traditional rural communities. The concepts of togetherness and cooperation cut across all of the themes identified in the results section. Both male and female participants emphasised working together and sharing housework for the benefit of the household. This concept of working together to provide food for the family is particularly important for policy. For the most part, policies have overlooked cooperation, yet the 1994 Cairo Declaration [25] emphasises cooperation between men and women. By emphasising women, policies are not taking advantage of the positive changes in men’s role. Policymakers can leverage on the cooperation between men and women to improve food security as well as gender outcomes.

The results of this study challenge the notion that women in developing countries are primarily responsible for producing, preparing and purchasing food. The data suggest that at least in this community, there is greater cooperation between men and women, especially when women are pregnant. The study supports other literature that indicates that men and women farm together (Doss, 2017). It also is consistent with literature suggesting that targeting both men and women can result in greater gender equality (Ragasa et al., 2019).

### Limitations and strengths

This study was conducted in only one district in rural central Malawi. While men’s involvement interventions have implemented in several other districts by NGOs and the Ministry of Health, this specific district was selected because of the significant increase in joint decision making observed in the 2015–2016 Malawi Demographic and Health Survey [42]. The sampling of only one community is recognised as a limitation. However, working with one community enabled us to conduct an in-depth study of men’s involvement in maternal and child health. Our findings help us to fill the gap in literature on men's participation in women's and children's health and could be used to inform policy in other sub-Saharan African countries with similar contexts.

## Conclusions and recommendations

The policy chronology indicates that men’s involvement in maternal and child health approaches in Malawi have been integrated into health policies as well as nutrition policies. Respondents reported that interventions in the health sector that have persuaded men to take a more active role in maternal and child health have contributed to changes in gender dynamics at the community level. While these changing gender dynamics reflect the perception of the focus group discussants, opportunities exist for other sectors such as agriculture to leverage on cooperation between men and women to improve food production, access to food as well as the nutrition of household members. This study provides evidence for the need for increased collaboration across sectors because of the impact that interventions implemented in one sector can have on gender relations at the community level.

Cross-cutting issues such as food security and gender could increase intra-governmental cooperation.

The nutrition policy integrated the changing gender roles because of the intersections between health and nutrition. Nutrition and gender are issues that cut-across the health, agriculture and other sectors. Cross-cutting issues such as food security, nutrition and gender could offer bridges for ensuring coordination and collaboration between the sectors.

Gender relations are not static. Policymakers should continuously assess the gender situation before designing policies. As the transversal development strategy document, the Malawi Growth and Development Strategy III should provide guidance on gender approaches, highlighting the importance of assessing the gender situation at the community level. The Strategy should promote coordination through collaboration amongst different stakeholders within and outside of government.

Targeting men can increase cooperation between men and women. Policies need to foster cooperation between men and women to maximise the potential of men and women in meeting food security and nutrition objectives. These policies need to be designed in a manner that harnesses the complementarity of the two sexes.

## Supporting information

S1 File Semi-structured interview guide. (DOCX)

S2 File Mndandanda wofunsira mafunso ochepa. (DOCX)
