# Supplementary material for: “The role of the man is to look for food”: Lessons from men’s involvement in maternal and child health programmes in rural Central Malawi
Source: PLoS One. 2019 Aug 23;14(8):e0221623. doi: 10.1371/journal.pone.0221623 (PMC6707577; doi:10.1371/journal.pone.0221623)
Supplement: S1 File — (DOCX) [file pone.0221623.s001.docx]

## Semi-structured interview guide English

My name is Elizabeth Mkandawire. I am from the University of Pretoria in South Africa. The purpose of this study is to understand interpretations of gender and how they influence nutrition policy. The information gathered is for my studies and will also be used to develop research policy briefs to improve nutrition policies. The questions I will ask relate to your knowledge in the area of nutrition/gender. You have been selected because you are in a position to provide information that is valuable to the study. Your participation in this study is voluntary and you may choose to withdraw at any time.

**Name of respondent:**

1. **Agenda-setting**

How are men involved in maternal and child health?
Do men help with cooking?
Do they help with any other domestic duties?
What was the trigger for this change?

1. **Design**

What motivates men to participate in maternal and child health?
How have socio-cultural barriers been negotiated?
What is gender?

1. **Decision making**

How have the traditional authorities assisted in getting men to be involved in maternal and child health?

1. **Implementation**

Who have been the mains advocates of men’s involvement?
Is this happening in other areas?

1. **Evaluation and reform**

What do you think about the by-laws that are passed by the traditional authorities?

**Other noteworthy insights:**
