# Supplementary material for: “The role of the man is to look for food”: Lessons from men’s involvement in maternal and child health programmes in rural Central Malawi
Source: PLoS One. 2019 Aug 23;14(8):e0221623. doi: 10.1371/journal.pone.0221623 (PMC6707577; doi:10.1371/journal.pone.0221623)
Supplement: S2 File — (DOCX) [file pone.0221623.s002.docx]

## Mndandanda wofunsira mafunso ochepa

*Dzina langa ndine Elizabeth Mkandawire. Ndachokela ku yunivesite ya Pretoria ku South Africa. Cholinga cha kafukufukuyu ndikumvetsa katanthauzidwe ka jenda ndinso momwe nkhani za jenda zimakhudzira ndondomeko ya zakudya. Zotsatira za kafukufukuyu zitithandiza kumvetsa mmene tingakhazikitsire ndondomeko zotsatila pofuna kukhazikitsa ndondomeko ya zakudya. Mafunso omwe ndidzifunsa ndi okhudzana ndi chidziwitso chanu pankhani ya zakudya komanso jenda. Mwasankhidwa chifukwa muli ndi mwayi wopereka chidziwitso chomwe chili chofunika pa kafukufuku uyu.* *Kulowa nawo mu kafukufukuyu ndi mwaufulu ndipo mungasankhe kuchoka nthawi iliyonse.*

**Dzina la wovomera:**

1. **Agenda-setting**

*Azibambo akutengapo gawo lanji pa nkhani ya uchembele wabwino?*

*Amuna amathandizira kuphika?*
*Kodi amathandiza pa ntchito zina zapakhomo?
Chinali chiyani chomwe chinayambitsa kusintha uku?*

1. **Design**

*Nchiyani chimalimbikitsa amuna kutenga nawo mbali paUchembele yabwino?*

*Kodi pa nkhani ya jenda ndi chikhalidwe zakhala zikugwirizana bwanji?*

*Jenda imathantauza chiyani?*

1. **Decision making**

Kodi aTA amathandizira bwanji kuti abambo atengepo mbali pankhani ya uchembele wabwino

1. **Implementation**

*Ndi ndani amene amalimbikitsa kulowerera kwa amuna pankhani ya uchembele wabwino?
Kodi izi zikuchitika kumadera ena?*

1. **Kufufuza ndi kusintha**

Mukuganiza bwanji za malamulo omwe aperekedwa ndi akuluakulu apamwamba?

**Mfundo zina zofunikira:**
